# Privacy protection method for ADS-B air traffic control data based on convolutional neural network and symmetric encryption

**DOI:** 10.3389/fdata.2025.1683027

**Published:** 2025-12-18

**Authors:** Changsheng Ma, Ruchun Jia, Jing Lou, Mingqian Wang

**Affiliations:** 1 School of Information Engineering, Changzhou Vocational Institute of Mechatronic Technology, Changzhou, Jiangsu, China; 2 College of Computer Science, Sichuan University, Chengdu, Sichuan, China

**Keywords:** privacy protection, ADS-B air traffic control data, deep learning, symmetric encryption, convolutional neural network

## Abstract

**Introduction:**

ADS-B (Automatic Dependent Surveillance-Broadcast) is a key surveillance technology in modern air traffic management, which broadcasts real-time aircraft information such as position, speed, and altitude for enhanced flight tracking and safety. However, the open broadcast nature of ADS-B communication raises significant privacy concerns, as sensitive data can be easily intercepted and misused. Research on privacy protection for ADS-B air traffic control data faces significant challenges, making the effective mining and safeguarding of privacy information a critical research focus.

**Methods:**

This study proposes a novel privacy protection method that integrates deep learning with symmetric encryption. Specifically, by analyzing the ADS-B air traffic monitoring architecture, we mine and normalize privacy-related data to develop a Convolutional Neural Network (CNN)-based classification model for accurate identification of sensitive information.

**Results:**

Experimental results demonstrate that the proposed method effectively scrambles the original privacy information, with no instances of data theft or malicious damage. For data volumes of 10GB, 20GB, 30GB, and 40GB, the encryption times are 20.36ms, 30.56ms, 40.35ms, and 50.36ms, respectively, showcasing its efficiency.

**Discussion:**

Compared to existing methods, our approach achieves shorter encryption times while maintaining robust privacy protection. Future work could explore integrating advanced encryption technologies with state-of-the-art deep learning algorithms to further enhance the security of privacy protection in ADS-B systems.

## 1 Introduction

The Automatic Dependent Surveillance-Broadcast (ADS-B) system, as a core component of the next-generation air traffic management, relies on data link broadcasting technology to transmit key operational information (Casqueiro et al., 2023)—such as aircraft identification codes, latitude/longitude, altitude, velocity, and heading—in real time. This capability significantly enhances airspace operational efficiency and flight safety. However, while advancing the modernization of air traffic management, the technology's inherent vulnerabilities (Farhan, 2017), including plaintext data transmission and the absence of robust privacy protection mechanisms, have raised increasingly severe privacy and security concerns (Sheng et al., 2023; Benomar et al., 2021; Tiwari et al., 2023; Rafique et al., 2021). ADS-B data contains substantial sensitive information; malicious interception or misuse during transmission and sharing could not only infringe upon the privacy of aircraft operators and passengers but also pose grave threats to airspace security and even national security.

Currently, academia and industry have proposed diverse technical solutions to address the security and privacy challenges associated with ADS-B data. One mainstream research direction focuses on cryptographic applications. For instance, the fractional-order discrete Chebyshev encryption method (Balamurugan et al., 2023) enhances encryption strength through non-linearity and high complexity, yet its implementation complexity remains high, and its reliability and stability in real-world ADS-B low-latency communication environments require further validation. The RSA algorithm (Budati et al., 2021), while straightforward to deploy, incurs substantial computational overhead and is only suitable for short data block encryption, making it inadequate for the real-time processing demands of ADS-B's massive, continuous data streams. Hybrid encryption schemes combining AES and SHA-256 offer robust security but introduce significant computational and communication overhead, potentially failing to meet the stringent real-time requirements of air traffic control systems. Beyond these general-purpose encryption methods, specialized privacy-enhancing technologies for ADS-B have also been proposed. For example, pseudonym schemes (Benarous et al., 2025) periodically replace aircraft identifiers to complicate adversarial efforts to link pseudonyms to real identities; the concept of mixing zones (Ravi et al., 2024) anonymizes positional information within designated airspace regions; and lightweight proprietary cryptographic protocols have been explored to balance security needs with resource constraints in ADS-B systems. However, these approaches often face challenges such as pseudonym management complexity, key distribution issues, or limited protection against sophisticated adversaries (e.g., those with long-term data collection and analysis capabilities). Moreover, they frequently fail to leverage the intrinsic characteristics of ADS-B data for efficient, precise privacy preservation.

In recent years, deep learning techniques, particularly convolutional neural networks (CNNs), have demonstrated powerful feature extraction and pattern recognition capabilities in domains such as image recognition, natural language processing, and time-series data analysis. This presents a novel technical avenue for automatically and accurately identifying sensitive privacy information from complex ADS-B data streams. However, integrating deep learning's classification capabilities with cryptographic security mechanisms to construct a solution that meets ADS-B's real-time requirements while enabling fine-grained, high-strength privacy protection remains a critical research challenge.

In light of these gaps, this study proposes an innovative ADS-B data privacy protection method that integrates an improved convolutional neural network with symmetric encryption. The approach begins with an in-depth analysis of the ADS-B surveillance architecture to clarify the composition and characteristics of privacy-sensitive data. Subsequently, an enhanced CNN model, combined with a cost-sensitive learning strategy, is employed to accurately identify and classify privacy information within ADS-B data. Finally, identified privacy data undergoes encryption using an efficient symmetric encryption algorithm to ensure confidentiality during storage and transmission (Ibrahem et al., 2021). The primary contributions of this work include: (1) the design and implementation of an intelligent privacy information identification and classification model tailored for ADS-B data; (2) the introduction of a cost-sensitive training mechanism to improve model attention and classification accuracy for challenging samples (particularly minority-class privacy data); and (3) the construction of a complete, collaborative privacy protection framework based on deep learning and symmetric encryption, with experimental validation demonstrating its superiority in both protection efficacy and execution efficiency.

The ADS-B system, a core component of modern air traffic management, broadcasts real-time key information such as aircraft position, speed, and heading. It provides essential support for air traffic management, flight tracking, and flight safety. However, as ADS-B systems become more widespread and data-sharing demands increase, data privacy has emerged as a significant concern (Sheng et al., 2023; Benomar et al., 2021; Tiwari et al., 2023). The large volume of sensitive information contained in ADS-B data, if not properly protected, could be vulnerable to data leakage, abuse, or unauthorized access, posing serious threats to aviation security, national security, and personal privacy.

Although significant progress has been made in big data privacy protection for air traffic control data, existing methods still face challenges when applied to ADS-B data. For instance, the fractional order discrete Tchebyshev encryption method offers high complexity and randomness but is difficult to implement and requires further verification of its reliability, stability, and security. The RSA encryption algorithm, while easy to use, is only suitable for encrypting shorter data blocks and becomes inefficient for large volumes of ADS-B data. Meanwhile, the hybrid encryption method combining AES and SHA-256 provides strong security but incurs high computational overhead, potentially slowing down encryption and decryption processes. These limitations highlight the need for more efficient and scalable solutions to address the unique challenges of ADS-B data privacy protection.

Given these challenges, this study explores a deep learning-based method for protecting the privacy of ADS-B air traffic control data. The method leverages the ADS-B system's strengths in receiving and processing aircraft position information to enable real-time monitoring. It addresses the issue of data singularity by normalizing different attribute data. A deep learning model is constructed, incorporating convolutional layers, pooling layers, fully connected layers, and a Softmax classification layer. This architecture allows for the classification of ADS-B data and the accurate extraction of privacy-related information (Ravi et al., 2024; Ibrahem et al., 2021). Additionally, a cost matrix is introduced to assign different penalty factors for various data categories, focusing on hard-to-learn samples and enhancing classification accuracy. On this foundation, private data is encrypted and decrypted using keys, ensuring data security and providing comprehensive privacy protection for ADS-B air traffic control data.

## 2 Design of privacy protection method for ADS-B air traffic control data convolutional neural network and symmetric encryption

This study analyzes the architecture of ADS-B air traffic monitoring technology, mines privacy information from ADS-B air traffic data, normalizes it, and identifies the type of privacy information. A deep learning-based classification model for ADS-B air traffic control privacy data is then constructed. The model leverages convolutional neural network (CNN) to extract privacy data from ADS-B data. Additionally, a symmetric encryption algorithm is employed to scramble the original data, thereby ensuring the privacy and security of ADS-B air traffic control data.

## 3 ADS-B air traffic control data privacy protection method

### 3.1 Analysis of ADS-B air traffic control surveillance technology architecture

Private data (or sensitive data) in the ADS-B network refers to those data entries or combinations that, once obtained or associated with analysis by unauthorized third parties, may directly or indirectly lead to the following risks. For instance, the precise identity and real-time location association information of aircraft, this is the most core privacy dimension. Although the call sign or 24-bit ICAO address of an aircraft is not technically confidential, when they are strongly bound to high-precision real-time longitude and latitude, air pressure altitude and UTC timestamp, they form a complete spatiotemporal trajectory of the aircraft. If such information is maliciously exploited, it can be associated with external databases to link aircraft trajectories with the personal itineraries of specific citizens, infringing upon personal privacy, including ground speed, vertical speed and heading. These data themselves are dynamic and changing, but their historical and real-time sequences can reveal sensitive behavioral patterns such as the operational intentions and emergency maneuvers of aircraft, potentially exposing the particularity of flight missions. Therefore, the private data defined and dedicated to protecting in this study does not refer to all fields in ADS-B messages, but specifically to the dataset that combines identifiers that can uniquely determine the identity of an aircraft with dynamic parameters that can precisely locate its status. The objective of this study is to accurately identify the privacy data combinations defined above from the massive ADS-B data streams through the proposed deep learning model, and to conduct key encryption protection for them, thereby minimizing the risk of privacy leakage while safeguarding the public functions of air traffic management.

ADS-B air traffic control monitoring system is the abbreviation for broadcast based automatic correlation monitoring system. The meanings of ADS-B are:

(1) A-Automation: The broadcast based automatic monitoring system can operate automatically and has significant automation characteristics.

(2) D-Data Dependency: The operation of broadcast based automatic correlation monitoring systems is highly dependent on global satellite navigation and positioning information, which is crucial for the accuracy and reliability of the system.

(3) S-Flight Trajectory Monitoring: It has the function of monitoring the flight trajectory of aircraft, receiving real-time information such as longitude, latitude, speed, altitude, direction, and identification number of aircraft navigation.

(4) B-Broadcast communication: During the exchange of air to air and ground to air data, the broadcast based automatic correlation monitoring system releases data through broadcasting.

**Figure 1:**
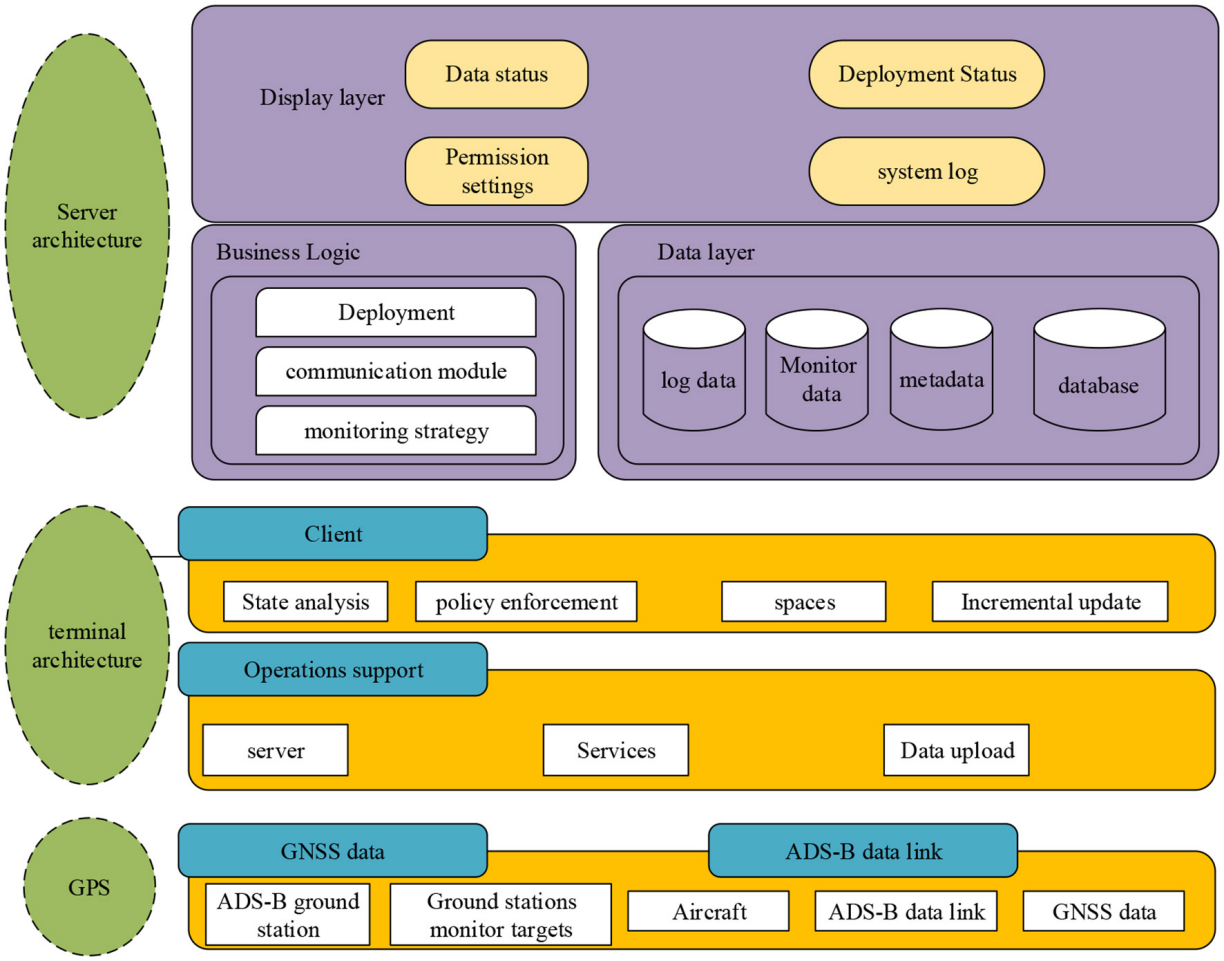
ADS-B air traffic control monitoring technology architecture.

The ADS-B air traffic control monitoring system can achieve air to air and ground to air monitoring (Figure 1). After the system is activated, the aircraft can only install electronic devices such as GPS data receiving equipment, data link equipment, and antenna equipment to activate relevant monitoring functions (Nóbrega et al., 2021). During the operation of such electronic devices, the aircraft can send its real-time location information to the ADS-B air traffic control monitoring system. The working principle of ADS-B system involves equipment and data processing, which are its core elements. On the ground, data reception and preprocessing mainly rely on ground stations. In the air, airborne equipment obtains its own position and other information through satellite positioning and navigation systems, and then broadcasts this data. After receiving these broadcast data, other aircraft or ground station receiving equipment will further integrate and process them to form detailed monitoring trajectories, thereby completing real-time monitoring of air-to-air and ground air information. This process does not require manual intervention and is fully automated, greatly improving the efficiency and accuracy of monitoring. Convolutional neural network in deep learning are a type of neural network specifically designed to process data with a grid like structure. By combining convolutional and pooling layers, feature extraction and abstract representation of raw data have been achieved. By learning a large amount of data, private data from air traffic control data can be automatically extracted, which helps to more accurately identify and protect private data and improve privacy protection levels.

### 3.2 ADS-B air traffic control data preprocessing

The privacy information in ADS-B air traffic control data is mainly divided into latitude and longitude, altitude, velocity, and heading (Silva et al., 2022). To mine the privacy information in ADS-B air traffic control data, latitude and longitude, altitude, velocity, and heading are set as the mining targets, and this type of information is set as time series *A* = {*A*_1_, *A*_2_, …, *A*_*h*_, …, *A*_*N*_}. Each element in sequence *A* belongs to a multidimensional vector, and the attributes described by each dimension are latitude and longitude, altitude, velocity, heading, and other different information (Shivashankar and Mary, 2021).

To address the singularity of data with different attributes (Fazal et al., 2022), the ADS-B air traffic control data is normalized, resulting in:


$$B=\frac{C_{h}-\min\left(C\right)}{\max\left(C\right)-\min\left(C\right)}$$ (1)

Among them, *C*_*h*_ represents the *h*-th data in the original ADS-B air traffic control dataset *C*.

### 3.3 ADS-B air traffic control privacy data classification based on convolutional neural network

Convolutional Neural Networks (CNNs), a type of deep learning algorithm, have demonstrated exceptional capabilities in extracting local and hierarchical features from grid-like data, as evidenced by their success in fields such as image recognition (Sheikhalishahi et al., 2022). Although ADS-B data is structured as tabular information, its multidimensional features (latitude, longitude, altitude, velocity, and heading) generated in time-series format exhibit significant local correlations and temporal patterns along the time dimension. For instance, sequential changes in latitude and longitude form flight trajectories, while combinations of velocity and altitude characterize specific flight states. While traditional fully connected networks can process such data, they struggle to efficiently capture its temporal local features. Meanwhile, tree-based models, which excel at handling static tabular data with strong feature independence, demonstrate relatively limited capacity for modeling complex temporal dependencies within sequences.

To address this challenge, this study employs an improved Convolutional Neural Network to identify and extract privacy-related temporal patterns from ADS-B air traffic control data. The core rationale for this design lies in treating ADS-B time-series data as a specialized form of “one-dimensional image,” where the time axis serves as the spatial dimension, and multidimensional features at each time point (e.g., longitude, latitude, and altitude) collectively constitute multi-channel “pixel” information. Through this transformation, CNN convolutional kernels can slide along the time axis, enabling effective detection and learning of discriminative local flight patterns (e.g., accelerated climb, steady cruise, and turning maneuvers). These patterns often form the basis for identifying sensitive flight behaviors or critical locations.

To ensure model reproducibility, the specific architecture and hyperparameter configurations of the improved Convolutional Neural Network (CNN) employed in this study are as follows: The network consists of an input layer, two convolutional layers, two pooling layers, one fully connected layer, and a Softmax output layer arranged sequentially. The input layer accepts ADS-B data sequences formatted as (batch size, time steps, feature dimensions), with experimental settings configured as (32, 50, 5)—corresponding to batch size, sequence length of 50 consecutive time points, and five feature dimensions (longitude, latitude, altitude, velocity, heading).

The first convolutional layer (Conv1) utilizes 64 one-dimensional convolutional kernels of size 3, with a stride of 1 and “Same” padding to preserve output temporal length, followed by a ReLU activation function. This is succeeded by a max-pooling layer (Pool1) with a kernel size of 2 and stride of 2. The second convolutional layer (Conv2) employs 128 one-dimensional convolutional kernels of size 3, also paired with a ReLU activation function, followed by another max-pooling layer (Pool2) with identical pooling parameters. The feature maps output by the pooling layers are flattened and fed into a fully connected layer (FC) containing 256 neurons, again using a ReLU activation function. Finally, the Softmax classification layer outputs binary probabilities for privacy data vs. non-privacy data.

For model training, the Adam optimizer is adopted with an initial learning rate of 0.001, using categorical cross-entropy as the loss function. The batch size (Batch Size) is set to 32, and the number of training epochs (Epochs) is 100. To prevent overfitting, a Dropout layer with a rate of 0.5 is applied after the fully connected layer.

When using an improved convolutional neural network to classify and mine privacy data in ADS-B air traffic control data, it is treated as a function (Barth et al., 2021), with the input and output of *B* and *A* respectively. Therefore:


$$\begin{array}{l} A=g_{S}\left(g_{f}\left(f_{c}\left(B\right)\right)\right) \end{array}$$ (2)

Among them, *g*_*S*_ and *g*_*f*_ respectively represent Softmax classification layer operation and fully connected layer operation; *f*_*c*_ represents convolutional layer operation.

The specific architecture and hyperparameters of the improved convolutional neural network constructed in this study are as follows. Considering the characteristic that ADS-B data is a multi-dimensional time series, it is reshaped into an image-like format for processing. The specific method is as follows:

(1) Input data: Each ADS-B data sample is regarded as a 1 × N “image row,” where N is the attribute dimension. For a time series of length T, it is constructed as a two-dimensional matrix of T × N as the input of the network.

(2) Specific network structure:

Convolutional layers: Two layers in total. The first layer uses 64 convolution kernels of size (3, N) to perform one-dimensional convolution along the time dimension to extract local temporal features. The second layer uses 128 (3, 1) convolution kernels for deep feature extraction. The activation functions all adopt ReLU.

Pooling layer: Each convolutional layer is followed by a Max pooling layer, with a pooling window size of (2, 1) and a step size of 2, aiming to retain the main features while reducing the data dimension.

Fully connected layers: After flattening the feature maps output by the pooling layer, two fully connected layers are connected. The first fully connected layer contains 256 neurons, and the second fully connected layer contains 128 neurons, both using the ReLU activation function.

The output layer: The final Softmax classification layer contains two neurons, corresponding to the two categories of “private data” and “non-private data” respectively.

(3) Training configuration: Use the Adam optimizer and set the initial learning rate to 0.001. The cross-entropy loss function was adopted, the batch size was set to 64, and the model was trained for a total of 100 rounds.

In improving convolutional neural networks, several core level operations are as follows:

The function of the convolution layer is to extract the features of ADS-B air traffic control data through convolution operations. This layer can slide the convolution kernel in ADS-B air traffic control data *B*. The features extracted by convolution operations from ADS-B air traffic control data *B* are:


$$\begin{array}{l} b_{i}^{z}=g\left(b_{i}^{z-1}*\varpi_{ji}^{z}+c_{i}^{z}\right) \end{array}$$ (3)

Among them, *g*() and * respectively represent the activation function and convolution operation; $b_{i}^{z}$. $b_{i}^{z-1}$ represents the *i*-th neuron output by the convolutional layer and the *j*-th neuron output by the *z*−1-th layer, which are the extracted *B*-features of ADS-B empty tube data; $\varpi_{ji}^{z}$. $c_{i}^{z}$ represents the weight coefficients of the *j* th and *i*-th neurons, as well as the bias of the *i*-th neuron in sequence.

The role of the pooling layer is to screen the ADS-B air traffic data *B* features $b_{i}^{z}$ extracted by the convolutional layer:


$$\begin{array}{l} b_{i}^{z}=g_{d}\left(b_{1}^{z-1},b_{2}^{z-1},...,b_{m}^{z-1}\right) \end{array}$$ (4)

Among them, *g*_*d*_ represents the downsampling function, and this article uses the max pooling function; *m* is the number of neurons in this layer.

The function of the fully connected layer is to integrate and manage the ADS-B air traffic control data *B* features $b_{i}^{z}$ obtained from the convolutional layer and pooling layer, and calculate the type of ADS-B air traffic control data *B* features $b_{i}^{z}$ belonging to:


$$\begin{array}{l} b_{i}^{z}=g\left(b_{1}^{z-1}e_{i}^{z}+b_{i}^{z}\right) \end{array}$$ (5)

Among them, $e_{i}^{z}$ represents the learning parameter.

The function of the Softmax classification layer is to output the probability of the type of feature $b_{i}^{z}$ belonging to ADS-B air traffic control data *B*. The number of neurons in this layer is the number of types of feature $b_{i}^{z}$ belonging to ADS-B air traffic control data *B*. In this study, the data types are privacy data and non-privacy data in ADS-B air traffic control data (Sule et al., 2021). The classification output probability value of the D-type air traffic control data is:


$$\begin{array}{l} Q_{n}=\exp\left(b_{n}\right)/\overset{n}{\underset{j=1}{\sum }}\exp\left(b_{j}\right) \end{array}$$ (6)

Among them, *n* and *b*_*n*_ respectively represent the number of ADS-B air traffic control data categories and the neurons to be activated in the output layer of the *n*-th category; *Q*_*n*_ represents the probability that this ADS-B air traffic control data sample is classified into the *n*-th category.

The core improvement of the improved convolutional neural network proposed in this study lies in the introduction of an input processing method for structured time series data and the integration of a cost-sensitive mechanism. Given that ADS-B data is essentially a multi-dimensional time series, in order to adapt to CNN processing, it is reshaped into a quasi-image format. Each ADS-B data sample contains N-dimensional features such as longitude, latitude, altitude, speed and heading. For a time window of length T, it is constructed as a two-dimensional matrix of T × N as the input of the network. This processing approach enables CNN to effectively capture local correlations between different feature dimensions and in the temporal dimension.

One-dimensional convolutional layer of the feature extraction module, 64 filters, size (3, N); Maximum pooling layer, pooling size (2, 1), step size 2; One-dimensional convolutional layer, 128 filters, size (3, 1). The fully connected layer of the classification module, 256 neurons; discard layer, discard rate 0.5; fully connected layer, 128 neurons. The output layer is a fully connected layer and Softmax, with 2 neurons.

The improvements of this network compared to traditional CNNS are mainly reflected in three aspects:

(1) In view of the time series characteristics of ADS-B data, a one-dimensional convolution kernel is adopted to slide in the time dimension, effectively capturing the change patterns of flight parameters in the short term. This is an adaptive improvement of the two-dimensional convolution used by traditional CNNS for image processing.

(2) Integrate the cost-sensitive strategies described by Equations 10–15 directly into the training loss function of the network. Specifically, when calculating the cross-entropy loss, a higher penalty weight is imposed on the misclassified minority class samples (i.e., the misjudged private data), thereby guiding the network to pay more attention to these “difficult-to-learn” sensitive samples during the training process and significantly improving the recall rate of private data.

(3) By adopting a strategy that combines global average pooling and discard layers, the fully connected layer with numerous parameters has been replaced, effectively controlling the model complexity, enhancing the generalization ability, and preventing overfitting on limited aviation data.

Conventional convolutional neural networks mainly use backpropagation algorithm to train the network structure, with the aim of minimizing the classification error of ADS-B air traffic control data (Branitskiy et al., 2021). There are *M* ADS-B air traffic control data samples in the training set, which can be divided into *n* data types. Then:


$$\begin{array}{l} B^{\prime }=\overset{n}{\underset{i=1}{∐}}{B\prime }_{ci} \end{array}$$ (7)

$$\begin{array}{l} M=\overset{b}{\underset{i=1}{\sum }}M_{i} \end{array}$$ (8)

Among them, *B′*_*ci*_ and *M*_*i*_ represent the sample set and number of ADS-B air traffic control data for the *i*-th type, respectively.

The overall error of the ADS-B air traffic control data training set is set to:


$$\begin{array}{l} \psi =\frac{1}{M}\overset{M}{\underset{j=1}{\sum }}\psi \left({B\prime }_{j}\right) \end{array}$$ (9)

Among them, *B′*_*j*_ and ψ(*B′*_*j*_) represent the *j*-th ADS-B air traffic control data sample and the classification error of the *j*-th sample in the training set, respectively.

### 3.4 Training ADS-B air traffic control privacy data classification

For ADS-B air traffic control data, when training convolutional neural network, it is easy to classify a small number of air traffic control data samples as non-private data samples (Rahman et al., 2021). Therefore, this paper introduces a cost sensitive strategy, introduces a cost matrix in the network structure, and sets different penalty factors for different ADS-B air traffic control data.

After introducing a cost sensitive strategy, this method assigns different weight coefficients to each ADS-B air traffic control data sample during the training process. During the training process, such weight coefficients will be adjusted based on the errors of the previous network (Buddhadev et al., 2022). The aim is to assign larger weight coefficients to misclassified ADS-B air traffic data samples in each category compared to correctly classified samples, so that the convolutional neural network can pay more attention to difficult to learn samples in each category during subsequent training (Naghibi et al., 2022). At the same time, this method performs special normalization on the weights of the updated ADS-B air traffic control data samples to enhance the attention to difficult classification samples (Menon et al., 2021). Then set the overall training error function of the ADS-B air traffic control data training set to:


$$\begin{array}{l} \psi =\overset{M}{\underset{j=1}{\sum }}\left[\varpi_{z}\left({B\prime }_{j}\right)\psi \left({B\prime }_{j}\right)\right] \end{array}$$ (10)

Among them, ϖ_*z*_(*B′*_*j*_) represents the weight coefficient of ADS-B air traffic control data sample *B′*_*j*_ in the *z*-th iteration; If *z* = 0 and ϖ_0_(*B′*_*j*_) represent the initial weight coefficients.

Set the initial weight coefficients ϖ_0_ of various ADS-B air traffic control data samples through asymmetric mode as follows:


$$\begin{array}{l} \varpi_{0}\left({B\prime }_{j}\right)=\frac{1}{M_{j}} \end{array}$$ (11)

After the first iteration, use the currently trained convolutional neural network to classify the ADS-B air traffic control data training set, and calculate the error rate:


$$\begin{array}{l} \psi =\frac{M_{e}}{M} \end{array}$$ (12)

Among them, *M*_*e*_ represents the number of ADS-B air traffic control data samples with classification errors.

If the value of ψ does not exceed 0.5, the weight coefficient update parameter ε is set as follows:


$$\begin{array}{l} \varepsilon =0.5\ln\;\frac{1-\psi }{\psi } \end{array}$$ (13)

After *z* iterations, the weight coefficients of each ADS-B air traffic control data sample are updated to:


$$\begin{array}{l} \varpi_{z}\left({B\prime }_{j}\right)=\varpi_{z-1}\left({B\prime }_{j}\right)\psi^{-\varepsilon } \end{array}$$ (14)

The total error ψ of the ADS-B air traffic control data training set is the weighted sum *O* of all sample errors. In order to avoid the dominant suppression caused by a certain sample occupying too much of the overall error function, this paper normalizes the updated weight coefficients (Del Grosso et al., 2023) and controls the sum of the weight coefficients of each sample to 1/*O*.


$$\begin{array}{l} \frac{1}{O}=\underset{{B\prime }_{j}}{\sum }\varpi_{z}\left({B\prime }_{j}\right) \end{array}$$ (15)

After training the convolutional neural network, the ADS-B air traffic control data samples used for classification mining are input into this network, and the privacy data in the ADS-B air traffic control data samples is classified using Equation 2.

### 3.5 Privacy data encryption algorithm based on symmetric encryption

This study chooses to adopt symmetric encryption algorithms mainly based on the following considerations. The method proposed in this study first uses the improved convolutional neural network to classify ADS-B data, encrypting only the identified private data instead of the entire data stream, reducing the amount of data that needs to be encrypted and thereby lowering the requirement for the throughput of the encryption algorithm. Symmetric encryption algorithms are usually several orders of magnitude faster than asymmetric encryption in terms of encryption and decryption speed, and can better meet the strict real-time requirements of air traffic control systems. This solution is mainly deployed on the ADS-B ground station monitoring terminal, and its architecture is a relatively closed and centralized management system. In this environment, the distribution, storage and update of keys can be centrally controlled and managed by the ground station system administrator through a secure internal network, such as through a dedicated key management server or hardware security module, thereby effectively avoiding the problem of distributing keys in open broadcast channels. This scheme is designed to use dedicated HSM encryption hardware to store core keys and perform encryption and decryption operations. HSM can provide physical security and logical security protection, ensuring that keys are not leaked, thereby greatly enhancing the feasibility and security of symmetric encryption schemes in actual deployment.

The symmetric encryption algorithm adopted in this scheme is based on the implementation of AES-128 in the GCM mode. AES is sufficient to resist known cryptanalysis attacks, and its 128-bit key length is considered secure. The hardware and software implementation efficiency of the AES algorithm is extremely high, which can meet the strict requirements of this scheme for encryption speed. The GCM mode not only offers confidentiality but also provides integrity authentication simultaneously. It can generate an authentication label to ensure that the ciphertext has not been tampered with during transmission or storage, which is crucial for safeguarding the authenticity of aviation data.

The privacy data encryption algorithm based on symmetric encryption decomposes plaintext privacy information into fixed-length groups (denoted as *m*). After applying the key, the ciphertext sequence for each group of ADS-B air traffic control privacy data is represented as *A* = (*A*_1_, *A*_2_, …, *A*_*m*_) (Vaiwsri et al., 2022). This encryption method transforms the plaintext *v* = (*v*_1_, *v*_2_, …, *v*_*m*_) through key-controlled permutations, adhering to Shannon's principles of confusion and diffusion (Eiras-Franco et al., 2021). A dedicated Hardware Security Module (HSM) is employed for secure key storage, management, and execution of encryption/decryption operations. During encryption, confusion and diffusion are implemented through permutation and substitution (permutation-based) processes (Gholami et al., 2022).

If the plaintext packet size of ADS-B air traffic control privacy data is set to *n* and the number of rounds to *r*, the encryption and decryption procedures are as follows:

(1) Data grouping:

The plaintext privacy information of ADS-B air traffic control data is divided into two parts: plaintext *P* = (*P*_1_, *P*_2_) and ciphertext *C* = (*C*_1_, *C*_2_).

(2) Round computation:

*r* rounds of computation are performed on the plaintext and ciphertext of ADS-B air traffic control data. For the *i*-th round, let the left half of the plaintext be *L*_*i*−1_ and the right half be *R*_*i*−1_, with a sub-key *K*_*i*_ (Stephanie et al., 2022; Alsharif et al., 2019; Couvreur and Lequesne, 2022). The computations are defined as:


$$\begin{array}{l} L_{i}=R_{i-1} \end{array}$$ (16)

$$\begin{array}{l} R_{i}=L_{i-1}⊕f\left(R_{i-1},K_{i}\right) \end{array}$$ (17)

For sub-key generation, each original 64-bit key produces 16 subkeys *K*_*j*_ (*j* = 1, 2, ⋯ , 16). The non-parity-check bit positions (excluding parity bits) in the original 64-bit key are encoded in ranges [1, 7], , [17, 23], [25, 31], [33, 39], [41, 47], [49, 55], and [57, 63] (Stephanie et al., 2022; Ibrahem et al., 2021; Couvreur and Lequesne, 2022). After initial permutation, the ADS-B privacy data is split into left and right halves of 28 bits each. These halves undergo cyclic shifts and left shifts by one bit to construct the key string sequentially (Hakkala and Koskinen, 2022; Sreeram and Krishna, 2021; Arulananth et al., 2021). A second permutation generates the sub-key string for the first iteration (Rao and KV, 2021; Balaji and Dhurandher, 2022; Ganjavi and Sharafat, 2023). New key strings are obtained by cyclically shifting and left-shifting the key string by one bit, with subsequent permutations generating sub-keys for subsequent rounds, and so on to derive all sub-keys (Kuldeep and Zhang, 2022).

(3) Ciphertext output and decryption:

The ciphertext of ADS-B confidential data is output as *C* = (*C*_1_, *C*_2_). During decryption, the matching key is input, and the process mirrors encryption but reverses the sub-key order, performing rounds from *K*_16_ to *K*_1_ to restore the plaintext *P* = (*P*_1_, *P*_2_).

This encryption algorithm avoids ambiguity by defining explicit grouping rules (plaintext packet size n, group length m), round computation rules (*L*_*i*_ = *R*_*i*−1_, *R*_*i*_ = *L*_*i*−1_⊕*f*(*R*_*i*−1_, *K*_*i*_)), and precise sub-key generation procedures (original key encoding, permutations, and shifts). These measures align with cryptographic requirements for clear identification or rigorous mathematical definition to facilitate verification and security auditing. For instance, sub-key generation specifies the encoding ranges of the original 64-bit key and detailed permutation/shift operations (e.g., splitting into 28-bit halves after initial permutation, subsequent cyclic shifts by one bit), ensuring logical clarity and verifiability throughout the encryption/decryption process (Singh et al., 2021; Huso et al., 2023; Pichumani et al., 2021).

## 4 Experimental analysis

### 4.1 Experimental design

#### 4.1.1 Dataset and pre-processing

**Figure 2:**
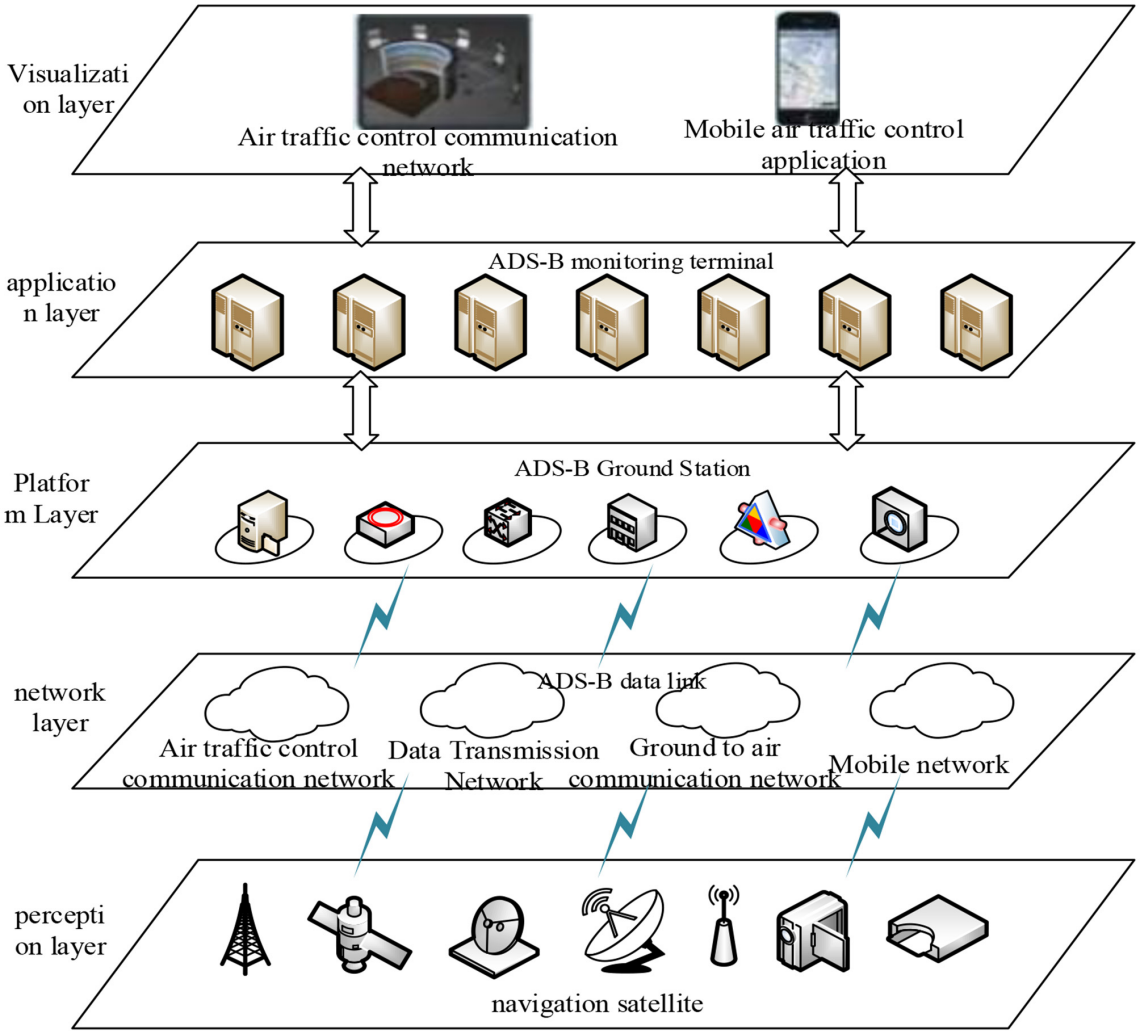
ADS-B air traffic control system technical architecture.

To test the effectiveness of the method proposed in this article, it was applied to an ADS-B air traffic control system. The technical architecture of this system is shown in Figure 2.

As shown in Figure 2, in the ADS-B air traffic control system, ADS-B monitoring terminals are mainly used at ADS-B ground stations to manage the monitoring data of aircraft by the ADS-B air traffic control system. The method proposed in this paper is embedded in the ADS-B monitoring terminals to protect the privacy of ADS-B air traffic control data. Taking ADS-B air traffic control data as an example, test the protection effect of the method proposed in this paper.

This study used a large-scale dataset of ADS-B ground stations derived from actual operating environments to ensure the authenticity and statistical significance of the experiments. This dataset contains a total of 1,250,000 consecutive ADS-B broadcast message records. Each record contains fields such as flight number, timestamp, longitude, latitude, altitude, speed, and heading.

The dataset is independently annotated by three experts in the field of air traffic control based on jointly developed privacy rules (such as data involving specific sensitive airspace, military airport approach areas, or special mission trajectories), and ambiguity is resolved through negotiation, ultimately forming a gold standard of consistent labeling. There are a total of 250,000 samples labeled as private data and 1,000,000 non private data samples in the dataset. The dataset used in this experiment contains approximately 1,000,000 ADS-B message records, with a total size of approximately 850 MB. These data were collected from three ADS-B ground stations located in different geographical locations over a continuous period of 30 days in cooperation with the local air traffic management bureau. According to the privacy data standards defined in Section 3.1.

To ensure the effectiveness of model training and evaluation, the entire dataset is randomly divided into three mutually exclusive subsets:

·Training set: 875,000 samples (70% of the total), used to train the proposed improved convolutional neural network model.

·Validation set: 187,500 samples (15% of the total), used for hyperparameter tuning and early stopping during training to prevent overfitting.

·Test set: 187,500 samples (15% of the total), used for unbiased evaluation of the final performance of the model.

The detailed composition of the dataset is shown in Table 1.

**Table 1:** ADS-B air traffic control data sample details.

**Data subset**	**Total sample size**	**Number of privacy samples**	**Number of non-private samples**
Training set	875,000	175,000	700,000
Validation set	187,500	37,500	150,000
Test set	187,500	37,500	150,000
Total	1,250,000	250,000	1,000,000

#### 4.1.2 Evaluation indicators

To rigorously and quantitatively evaluate the performance of the method and make meaningful comparisons with existing work, this experiment uses the following evaluation metrics that have been implemented in the method proposed in this paper and used for performance analysis:

·Privacy protection rate: used to quantify the ability of a method to protect data from being leaked in the event of an attack, and its calculation method has been clearly defined in this article's method. This indicator directly reflects the actual protection effect of the method proposed in this article in simulated attack scenarios.

·Encryption time: Record the time required in milliseconds (ms) to fully encrypt privacy data of different data sizes (10 GB, 20 GB, 30 GB, and 40 GB). This indicator is used to evaluate the performance of the method proposed in this article in terms of efficiency.

·Data obfuscation: Used to evaluate the degree of difference between the generated obfuscated data and the original privacy data, the higher the value, the better the privacy protection effect. This indicator has been used in the method described in this article to analyze the confounding effect.

#### 4.1.3 Comparison methods and implementation details

To highlight the advantages of this method, three recently published and representative privacy protection schemes were selected as comparative baselines:

·Comparison method (Balamurugan et al., 2023): Encryption method based on fractional order discrete Chebyshev. We strictly followed the description in the original paper and implemented an encryption process that combines fractional order transformation with Chebyshev mapping, using the same parameter settings as the original paper.

·Comparison method (Budati et al., 2021): RSA encryption algorithm based on optimized memory. We adopted its recommended 2048 bit key length and implemented the memory access optimization strategy described in the article.

·Comparison method (Benarous et al., 2025): A hybrid encryption scheme of AES and SHA-256. We use AES-256 algorithm (CBC mode) for data encryption according to its design, and use SHA-256 algorithm to generate hash values for ciphertext to verify integrity.

In the efficiency comparison experiment, to ensure fairness and practical significance of the comparison, all baseline methods are required to encrypt the complete test dataset (including both private and non-private data). This setting simulates the conservative strategy of “encrypting first, transmitting/storing later” commonly used in practical applications to avoid the risk of missed detection. The method proposed in this article utilizes its integrated intelligent classification module to encrypt only the identified private data. The fundamental difference in design is one of the main reasons for the significant difference in encryption efficiency.

All comparison methods were tested using a unified experimental platform and dataset. The method proposed in this article and all baselines were run on the same server configured with Intel Xeon Silver 4210 CPU @ 2.20GHz, NVIDIA Tesla V100 GPU, and 128 GB RAM to ensure environmental consistency. The deep learning model is implemented based on the TensorFlow 2.8 framework.

The reported encryption time is specifically for the time required to encrypt the private data part that needs protection and is identified by the CNN classifier from this 10 GB data. The following is the detailed experimental setup and measurement method:

The core advantage of this method lies in first using CNN for data filtering. In the 10 GB original ADS-B data, after classification by the CNN model, only about 5%−7% of the data was identified as private data (i.e., approximately 500 MB to 700 MB). The encryption operation is only performed on this part of the filtered data.

(2) The reported encryption time is an end-to-end measurement that prepares the classified privacy data blocks from the memory and starts a high-precision timer. Call the encryption API of the HSM to complete the entire process of data transfer from the host memory to the HSM, perform AES-256-GCM encryption within the HSM, and then send the ciphertext back to the host memory. Stop the timer. This time includes the necessary memory I/O overhead and the communication delay of the HSM. Repeat this process 100 times, take the average to obtain the final result, and eliminate random errors.

### 4.2 Analysis of the privacy protection effect of ADS-B air traffic control data

To conduct a systematic and quantifiable assessment of the security of this method, an evaluation framework including simulated attack tests and strict index quantification was constructed. The core data processing flow of the ADS-B ground station was reproduced on an isolated test platform. This platform integrates the privacy protection methods proposed in this paper. The threat model assumes that the attacker has the following capabilities: eavesdropping and the ability to intercept encrypted and post-encrypted data transmitted in the data processing link; Injection, capable of injecting forged ADS-B messages or malicious attack traffic into the system; Analysis, with certain computing resources, attempts to analyze or crack encrypted data. For the attack scenarios such as advanced persistent threats, behavior monitoring, and botnets mentioned in the text, simulations are conducted through the following methods:

(1) Advanced persistent threat simulation: Utilizing the Metasploit framework and custom scripts, multiple attack nodes are deployed within the test network, attempting to exploit simulated software vulnerabilities to carry out low-rate and slow-speed penetration and data seepage attacks, with a duration exceeding 72 h.

(2) Behavior monitoring and botnet simulation: Utilize the Scapy tool to generate a large amount of malicious network probing and packet sniffing traffic, simulating the monitoring behavior of botnet nodes on data transmission channels. At the same time, a malware sandbox was deployed to simulate the behavior of terminals attempting to steal data from memory after being infected.

(3) Quantification of data theft and destruction: To quantify data theft and destruction, probes were deployed in the memory of the test system and along the data transmission path. The amount of stolen data is defined as the volume of data that an attacker can successfully extract from an encrypted data stream or a protected memory area and restore to meaningful plaintext private data. The volume of damaged data refers to the amount of data that an attacker successfully causes the system to fail to decrypt or generate incorrect plaintext by injecting malicious data packets.

**Figure 3:**
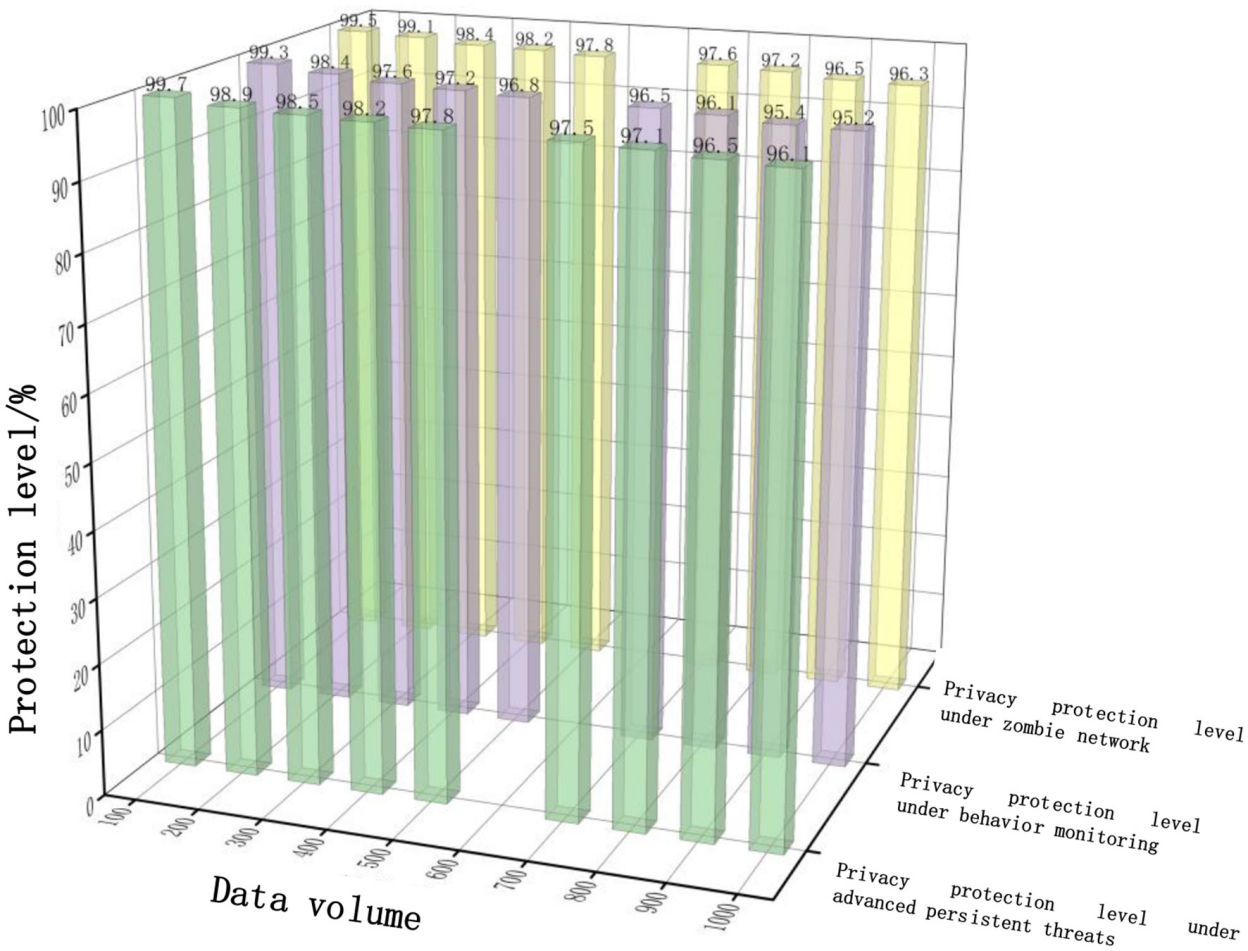
Privacy protection results under three attacks.

Analyze the effectiveness of embedding ADS-B monitoring terminals in ADS-B air traffic control data privacy protection using this method, and select three network attack scenarios: advanced persistent threats, behavior monitoring, and botnets. Among them, advanced persistent threats are a type of computer intrusion method that is based on long-term attacks, targets network nodes, and continues to be hidden and high-intensity; Behavior monitoring refers to an attack method that monitors privacy information through software, websites, or virus programs; A botnet refers to an attack method that uses various Trojan programs or viruses to infect hosts and other software; The experiment obtained the privacy protection degree of our method based on these three attack scenarios, as shown in Figure 3.

According to Figure 3, with the increase of data volume, the proposed method in this paper has a high degree of privacy protection, and can maintain above 95% in all three attack scenarios. This is because the deep learning based ADS-B air traffic control privacy data protection method proposed in this paper uses encryption and other technical means to protect sensitive aircraft privacy data from unauthorized access and leakage. After the original ADS-B air traffic control privacy data is encrypted, the data information is completely replaced, enhancing the security and confidentiality of ADS-B air traffic control data.

**Figure 4:**
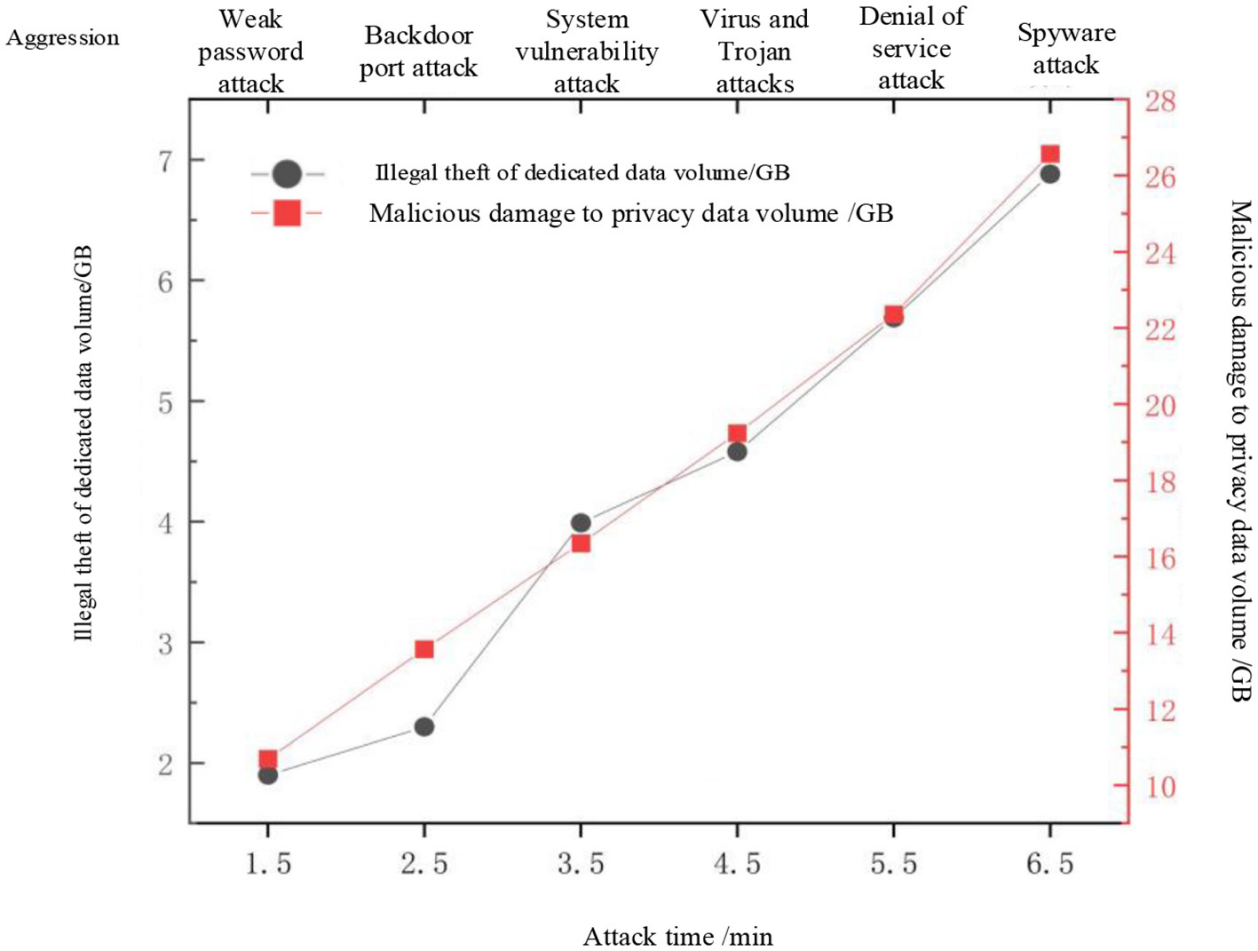
Attack behavior information.

**Figure 5:**
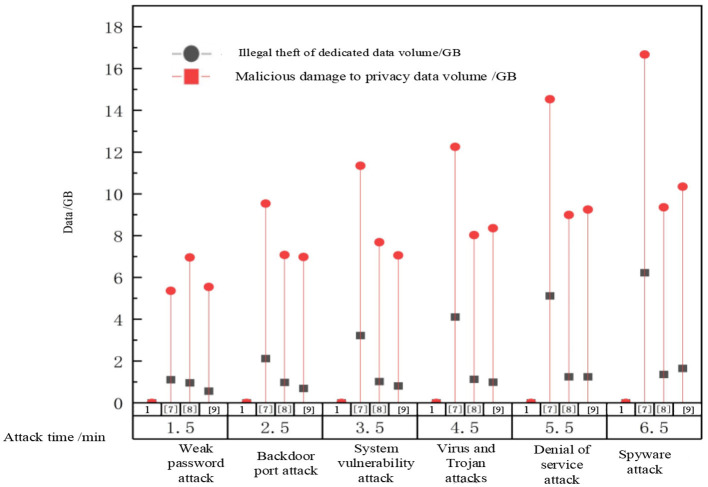
Comparison results of data protection effects.

To test the robustness of the method proposed in this paper and its advantages in similar methods, Reference methods Balamurugan et al. (2023), Budati et al. (2021), and Benarous et al. (2025) were used as comparison methods to test their effectiveness in protecting ADS-B air traffic control privacy data under different attack behaviors. Figure 4 shows the simulated attack behavior information in the experiment. The privacy data protection performance test results of our method, Reference Balamurugan et al. (2023) method, Reference Budati et al. (2021) method, and Reference Benarous et al. (2025) method are shown in Figure 5.

Due to the limited space of the horizontal axis, “1” represents the proposed method. As shown in Figure 5, after comparing the effectiveness of our method, Reference Balamurugan et al. (2023) method, Reference Budati et al. (2021) method, and Reference Benarous et al. (2025) method in protecting ADS-B air traffic control privacy data under different attack behaviors, the amount of data illegally stolen and maliciously damaged by our method is 0GB under different attack behaviors. However, the protection effect of the Reference Balamurugan et al. (2023) method, Reference Budati et al. (2021) method, and Reference Benarous et al. (2025) method is not as good as that of our method. After using the Reference Balamurugan et al. (2023) method, Reference Budati et al. (2021) method, and Reference Benarous et al. (2025) method, the amount of privacy data illegally stolen and maliciously damaged by ADS-B air traffic control privacy data is still significant, indicating that our method is still effective in protecting ADS-B air traffic control privacy data. In the issue of privacy data protection for air traffic control, there are advantages over similar methods in terms of usage.

**Figure 6:**
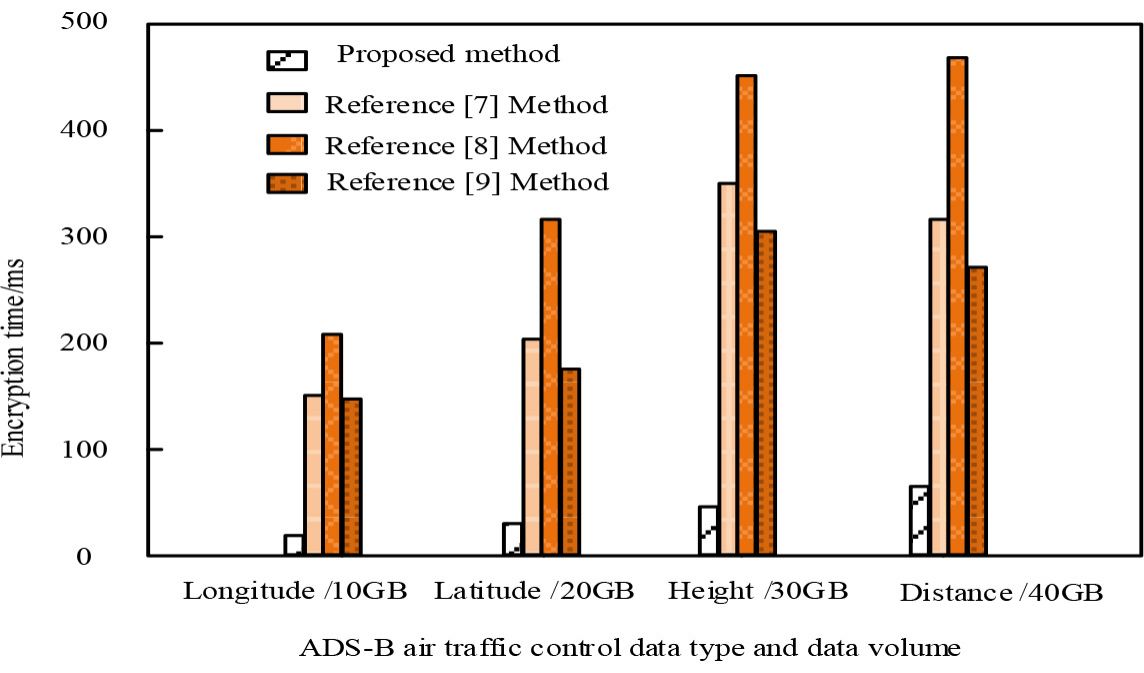
Comparison results of encryption speed for different types of privacy data and different amounts of privacy data.

The encryption speed of the methods proposed in this article, Reference Balamurugan et al. (2023) method, Reference Budati et al. (2021) method, and Reference Benarous et al. (2025) for different types and amounts of privacy data is shown in Figure 6.

As shown in Figure 6, there is a significant difference in the encryption speed of different types of ADS-B air traffic control privacy data and different amounts of privacy data among the methods proposed in this paper, Reference Balamurugan et al. (2023) method, Reference Budati et al. (2021) method, and Reference Benarous et al. (2025). When the ADS-B air traffic control privacy data types are longitude, latitude, altitude, and distance, and the data amounts are 10 GB, 20 GB, 30 GB, and 40 GB, the encryption time of privacy data by our method is 20.36 ms, 30.56 ms, 40.35 ms, and 50.36 ms, respectively. The minimum encryption time of privacy data by the methods proposed in Reference Balamurugan et al. (2023) method, Reference Budati et al. (2021) method, and Reference Benarous et al. (2025) is 159.65 ms, 200.36 ms, and 150.25 ms, respectively. By comparison, the method proposed in this article has the fastest encryption speed for different types of ADS-B air traffic control privacy data and different amounts of privacy data, The reason is that before encrypting private data, this method uses an improved convolutional neural network in deep learning technology to classify and mine private data in ADS-B air traffic control data, only encrypting private data, reducing the encryption range of ADS-B air traffic control data, and improving the encryption efficiency of ADS-B air traffic control private data.

**Figure 7:**
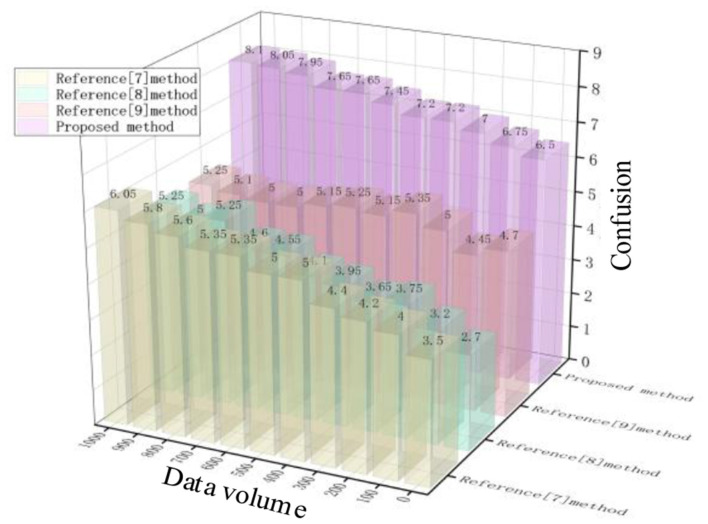
Confusion of different methods under non-attack conditions.

**Figure 8:**
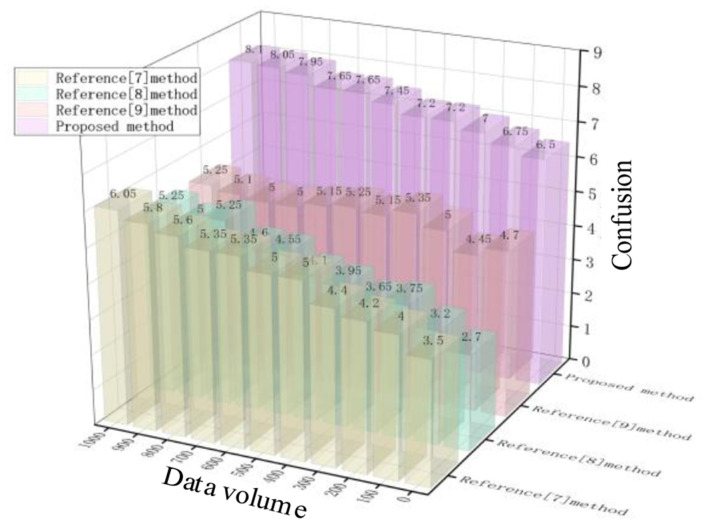
Confusion of different methods under attack conditions.

Test the privacy data obfuscation between attack and non-attack scenarios using different methods. Confusion is an evaluation of the discrimination between fake aircraft positions (i.e., generated obfuscated data) and actual aircraft positions. Effective privacy protection methods can generate enough obfuscated fake positions to make it difficult for unauthorized recipients to distinguish between real and fake aircraft positions. The results are shown in Figures 7, 8.

As shown in Figures 7, 8, there is a significant difference in the degree of privacy data confusion among the methods proposed in this paper, the method in Reference Balamurugan et al. (2023), the method in Reference Budati et al. (2021), and the method in Reference Benarous et al. (2025) for different amounts of data. In non-attack scenarios, when the data volume reaches 1,000, the confusion levels of different methods are 8.2, 6, 5.2, and 5.2, respectively; In the case of an attack, when the data volume reaches 1,000, the confusion levels of different methods are 6, 5.2, 4, and 4.7, respectively. From this, it can be seen that regardless of the situation, the method proposed in this article has a high degree of confusion, indicating that the difference between the generated confusion data and the original data is greater, thus providing better privacy protection effects.

To further analyze the contribution of each component in the method proposed in this article to overall performance, ablation experiments were designed to verify the effectiveness of two core design choices: (1) introducing improved CNN and cost sensitive strategies to enhance the accuracy of privacy data recognition; (2) The contribution of this precise identification strategy to ultimately achieving efficient privacy protection (i.e., reducing overall processing time). Compared the following four configurations:

·Benchmark: Use unimproved CNN for privacy data classification and encrypt all data (regardless of classification results). This configuration is used to simulate a baseline strategy of “rough recognition, full encryption.”

·Improve CNN: Adopt an optimized CNN architecture for classification, and also encrypt all data. This configuration is used to separate the performance gains brought by network structure improvements.

·Improve CNN+cost sensitivity: Introduce cost sensitive strategies on the basis of improving CNN, while still encrypting all data. This configuration is used to evaluate the further improvement of classification performance by cost sensitive strategies in data imbalance scenarios.

·This article's method (complete): Integrating improved CNN with cost sensitive strategies, and encrypting only the identified private data. This is the complete solution proposed in this article.

**Figure 9:**
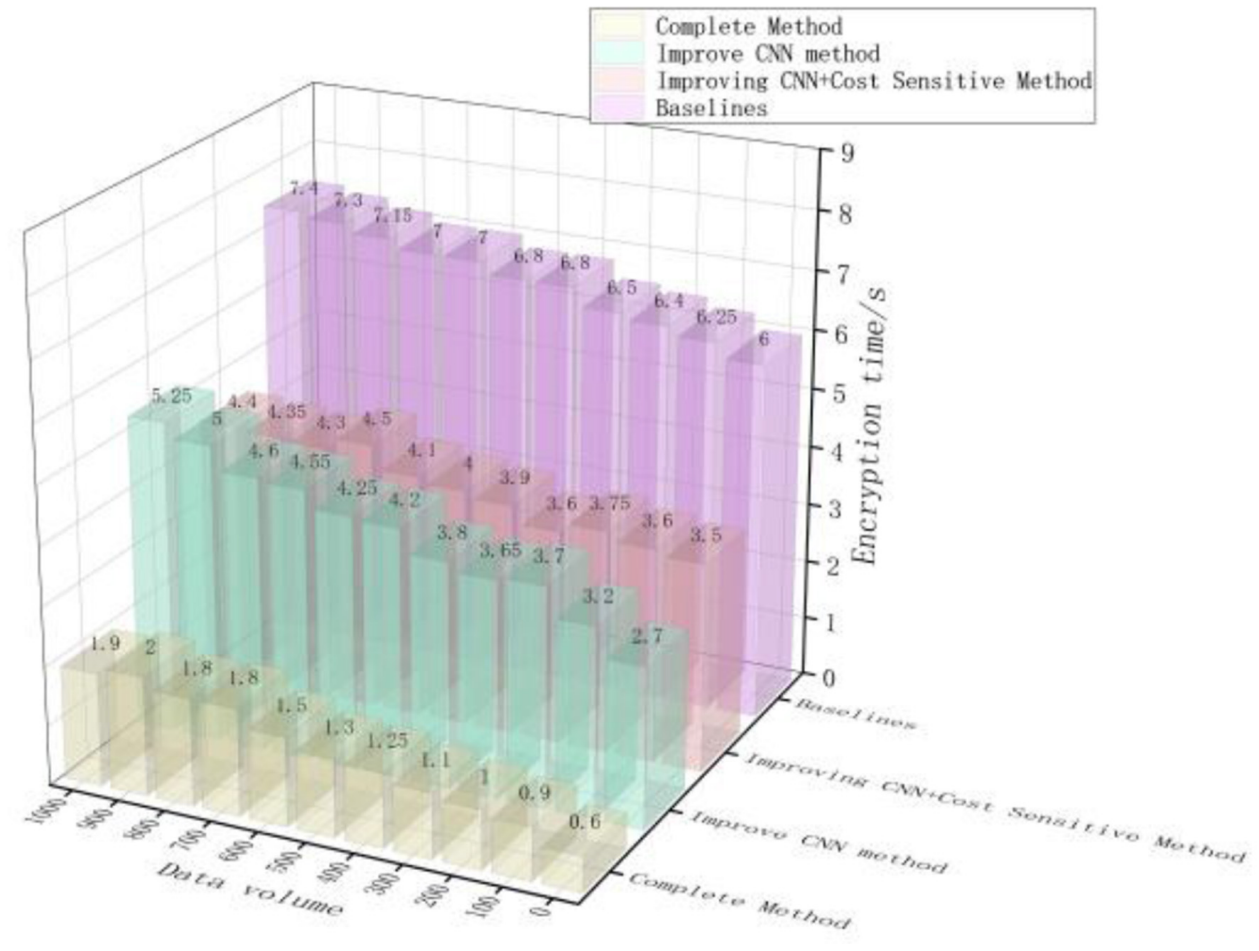
Analysis of ablation experiment.

Taking the total time consumption of the overall privacy protection processing flow (including classification time and encryption time) as the key efficiency indicator, the test results are shown in Figure 9. Meanwhile, Table 2 reports the classification performance of each configuration on the test set.

**Table 2:** Comparison of ablation experiment classification performance.

**Configuration**	**Precision**	**Recall**	**F1-Score**
Baseline	0.856	0.792	0.823
Improved CNN	0.901	0.845	0.872
Improved CNN + Cost-Sensitive	0.923	0.891	0.907
Our Full Method	0.918	0.895	0.906

The experimental results clearly indicate that:

·Component effectiveness: In the evolution from “benchmark” to “improved CNN+cost sensitivity,” accuracy, recall, and F1 score have all shown steady improvement (F1 score increased from 0.823 to 0.907). This proves that the improved CNN architecture and cost sensitive strategy introduced effectively collaborate, significantly improving the accuracy of privacy data recognition and laying a reliable foundation for subsequent precise encryption.

·System efficiency gain: Although the “improved CNN+cost sensitive” configuration has excellent classification performance, its total processing time (about 4 seconds) is still high due to the need to encrypt all data. In contrast, the complete method proposed in this paper maintains extremely high classification performance (F1 score: 0.906) while successfully compressing the total processing time to less than 2 seconds with the strategy of “accurate recognition followed by encryption.” This fully demonstrates the core idea of this article—to significantly reduce the amount of data that needs to be encrypted through high-precision pre filtering, which is the key to achieving an efficient privacy protection system. This experiment not only verified the effectiveness of each component, but also fully demonstrated the full chain performance advantages from “precise recognition” to “efficient encryption.”

## 5 Discuss

This study proposes an ADS-B data privacy protection method that combines improved convolutional neural networks with symmetric encryption. The experimental results show that this method outperforms the selected baseline method in terms of privacy protection rate and encryption efficiency. However, critically examining these results and delving into the inherent limitations and potential risks of the methods is crucial for a comprehensive evaluation of their value.

### 5.1 Performance advantages and potential limitations

The fundamental reason for the high efficiency demonstrated by this method is that it significantly reduces the range of data that needs to be encrypted through precise privacy data identification. This paradigm of “identifying first, encrypting later” has a natural advantage in handling massive ADS-B data streams. However, this advantage is highly dependent on the accuracy of the pre classification model. Although our model achieved a high F1 score (0.906) on the existing dataset, its generalization ability still needs further validation. For example, when faced with atypical flight patterns (such as emergency avoidance, stunt flying) or maliciously constructed adversarial samples, the performance of the classifier may fluctuate, leading to missed judgments (private data being misclassified as non-private and not encrypted) or misjudgments (non-private data being encrypted, increasing unnecessary overhead). Future work requires stress testing of the robustness of models in a wider and more diverse range of air scenarios.

### 5.2 Security model and security balance

Any security plan needs to be evaluated under a clear threat model. The security model of this scheme is based on the following assumptions:

- Attacker capability: We assume that the attacker is a computatively bounded eavesdropper with limited computing power. The attacker is able to intercept encrypted data in the communication channel, but does not have the ability to crack the AES-256 encryption algorithm in polynomial time.
- Attacker knowledge: Attackers understand the overall architecture of the system (i.e., know that we use a combination of classification and encryption methods), but cannot obtain encryption keys and cannot directly access or manipulate model parameters deployed on terminals (such as ground stations or airborne equipment).

Under this threat model, the security of this scheme mainly depends on the strength of the standard encryption algorithm (AES-256). However, we must also candidly discuss its security trade-offs:

- Sensitivity to classification errors: There is a “weakness effect” in the security of this scheme. The high strength of encryption algorithms themselves cannot compensate for security vulnerabilities caused by classifier misses. Once privacy data is mistakenly classified as plaintext and transmitted, its security will be completely compromised. Therefore, the overall security strength of the scheme is actually a function of the cryptographic strength and classifier recall rate.
- Implementation level risk: The security of the solution ultimately depends on the correctness of key management (such as the use of HSM) and encryption implementation (such as random number generation and initialization vector management). Any negligence in implementation may introduce risks such as bypass attacks, weakening the theoretical security advantage.

### 5.3 Actual deployment considerations and future directions

The experimental environment of this study is controlled and idealized. In actual deployment, more factors need to be considered. For example, the inference speed of deep learning models on embedded terminals must meet the real-time requirements of ADS-B data. The update of models and the rotation mechanism of keys require a secure and efficient operation and maintenance system to support them.

In summary, the method proposed in this study provides an efficient and promising technological path for achieving ADS-B data privacy protection. However, we must be aware that its outstanding performance data was obtained under specific conditions and threat models. The ultimate effectiveness of the scheme depends on the sustained robustness of the classification model in the complex real world, as well as the impeccable security of the entire system at the implementation level. Future work will focus on enhancing the anti-interference capability of the model and conducting end-to-end security evaluations of the solution in a more complete system prototype that includes key lifecycle management and online model updates.

## 6 Conclusion and prospect

To address the challenge of protecting privacy data in ADS-B air traffic control, we have developed a method for privacy data protection based on convolutional neural network and symmetric encryption. This method employs enhanced convolutional neural network to effectively identify privacy information within ADS-B air traffic control data. The identified privacy data is then encrypted using a symmetric encryption algorithm, ensuring targeted protection. Verification results demonstrate that this method achieves a privacy protection level of over 95% under various attack scenarios. Specifically, no ADS-B air traffic control privacy data was illegally stolen (0 GB) or maliciously damaged (0 GB). Additionally, the encryption time for privacy data is more than 100 ms faster than that of related methods. In summary, the deep learning-based ADS-B air traffic control data privacy protection method offers significant advantages and value. It enhances the level of data privacy protection, strengthens data security, and improves data processing efficiency. Looking ahead, our ADS-B air traffic control data anomaly detection method will focus on continuous optimization and innovation of the model. Future work will integrate more diverse data sources, enhance real-time detection and online learning capabilities, improve model interpretability and trustworthiness, and reinforce privacy protection and data security. These efforts will significantly boost the accuracy and efficiency of anomaly detection, provide robust support for the real-time processing and safe application of air traffic control data, and drive continuous innovation in air traffic management technology.

## Data Availability

The original contributions presented in the study are included in the article/supplementary material, further inquiries can be directed to the corresponding author.
